# Comparison of short axis and long axis acquisitions of T1 and extracellular volume mapping using MOLLI and SASHA in patients with myocardial infarction and healthy volunteers

**DOI:** 10.1186/s12880-019-0320-x

**Published:** 2019-02-22

**Authors:** Christos G. Xanthis, David Nordlund, Robert Jablonowski, Håkan Arheden

**Affiliations:** 1 Department of Clinical Sciences Lund, Clinical Physiology, Skåne University Hospital, Lund University, Lund, Sweden; 2 Laboratory of Computing, Medical Informatics and Biomedical – Imaging Technologies, School of Medicine, Aristotle University of Thessaloniki, Thessaloniki, Greece

**Keywords:** T1 mapping, Extracellular volume, Slice orientation, MOLLI, SASHA, Cardiovascular magnetic resonance

## Abstract

**Background:**

Although previous studies have examined the impact of slice position in volumetric measurements in Cardiovascular Magnetic Resonance (CMR) imaging, very limited data are available today comparing T1 and Extra-Cellular Volume (ECV) measurements from short and long axis acquisitions. The purpose of this study was to investigate the impact of slice position and orientation on T1 and ECV measurements using the MOdified Look-Locker Inversion recovery (MOLLI) and Saturation recovery single-shot acquisition (SASHA) sequence in patients with myocardial infarction and in healthy volunteers.

**Methods:**

Eight (8) healthy volunteers with no medical history and eight (8) patients with myocardial infarction were included in this study. MOLLI and SASHA were utilized and short-axis and long-axis images were acquired. T1 and ECV measurements were performed by drawing same size regions of interest on the myocardium as well in the blood pool at the intersections of the short axis and long axis images.

**Results:**

In healthy volunteers, there were no statistically significant differences in native T1 and ECV values between short axis and long axis acquisitions using MOLLI (two-chamber, three-chamber and four-chamber) and SASHA (three-chamber). In patients, there were no statistically significant differences in native T1 and ECV values between short axis and 3-chamber long axis acquisitions in both remote and affected myocardium using MOLLI and SASHA.

**Conclusions:**

Long axis measurements of myocardial T1 and ECV using MOLLI and SASHA exhibit good agreement with the corresponding short axis measurements allowing for fast and reliable myocardial tissue characterization in cases where shortening of the overall imaging acquisition is required.

## Background

In the field of Cardiovascular Magnetic Resonance (CMR) imaging, quantitative measures of myocardial and blood T1 before and after contrast injection enabled the calculation of myocardial extracellular volume fraction (ECV), an important myocardial biomarker [1, 2]. Several studies have demonstrated the potential of ECV measurement in the assessment of a variety of myocardial pathologies [3, 4] and in the guidance of therapy [5].

Myocardial and blood T1 values are most often determined on short-axis images of the left ventricle of the heart, usually including one apical, one midventricular and one basal slice. Recent advancements in the field of cardiac T1 mapping have allowed for fast generation of a single-slice T1 map within a single breath-hold acquisition. Today, the most commonly used T1 mapping technique in CMR is the MOdified Look-Locker Inversion recovery (MOLLI) [6] whereas the Saturation recovery single-shot acquisition (SASHA) [7] T1 mapping technique has been proposed as a means of mitigating the T1-underestimation in MOLLI [7, 8].

It has previously been shown that several factors affect the performance of MOLLI and SASHA on T1 and ECV mapping in terms of accuracy and precision [9]. Different T1 mapping methods and parameter sets result in different ranges of T1 and ECV values for normal myocardium and blood [8, 10, 11]. Although previous studies have examined the impact of slice position in measuring other cardiac parameters in CMR imaging (such as left ventricular mass, volume and ejection fraction) [12–14], very limited data are available today comparing T1 [15] and ECV measurements from short and long axis acquisitions [16, 17]. Despite this, current recommendations on T1 and ECV mapping in patients with global/diffuse cardiac disease [18] suggest adding a long axis map to aid analysis. Therefore, the specific aim of this study was to investigate the impact of slice position and orientation on T1 and ECV measurements. MOLLI and SASHA sequences were utilized in healthy volunteers and in patients with myocardial infarction to investigate the impact of slice position and orientation on T1 and ECV measurements.

## Methods

Eight (8) healthy volunteers with no medical history (5 men, 3 women, age 25 ± 5 years) and eight (8) patients (7 men, 1 woman, age 66 ± 10 years) with myocardial infarction and without any renal impairment were included in this study. The study was approved by the regional ethics committee and all subjects provided written informed consent (The regional ethics committee, Lund, Sweden. Ethics applications numbers: 541/2004 and 815/2016). Blood for hematocrit analysis was sampled from a peripheral vein from the subjects approximately 30 min after lying down, just before Gd-contrast administration.

### MR protocol

All subjects underwent CMR on a MAGNETOM Aera 1.5 T scanner (Siemens Healthcare, Erlangen, Germany) using a 30-channel coil (body array and spine array). In healthy volunteers, a prototype MOLLI sequence was used to acquire a single midventricular short-axis image and three long-axis images (two-chamber, three-chamber and four-chamber) whereas a prototype SASHA sequence was used to acquire a midventricular short-axis image and a single long-axis (three-chamber) image. Pre-contrast MOLLI T1 mapping was performed using an acquisition scheme of 5s(3s)3s whereas post-contrast MOLLI T1 mapping was performed using an acquisition scheme of 4s(1s)3s(1s)2s. The SASHA scheme remained the same before and after contrast administration. Previous studies [7, 8] have shown that these pulse sequences are heart-rate independent. Post-contrast mapping was performed approximately 30 min after injection of 0.2 mmol/kg Gd-DOTA (Dotarem, Guerbet, Roissy, France). In patients, the same MR protocol was utilized for the acquisition of a single midventricular short-axis image and a single long-axis image (two-chamber or three-chamber). MOLLI and SASHA T1 maps were acquired at the same slice locations.

### Image analysis

**Fig. 1:**
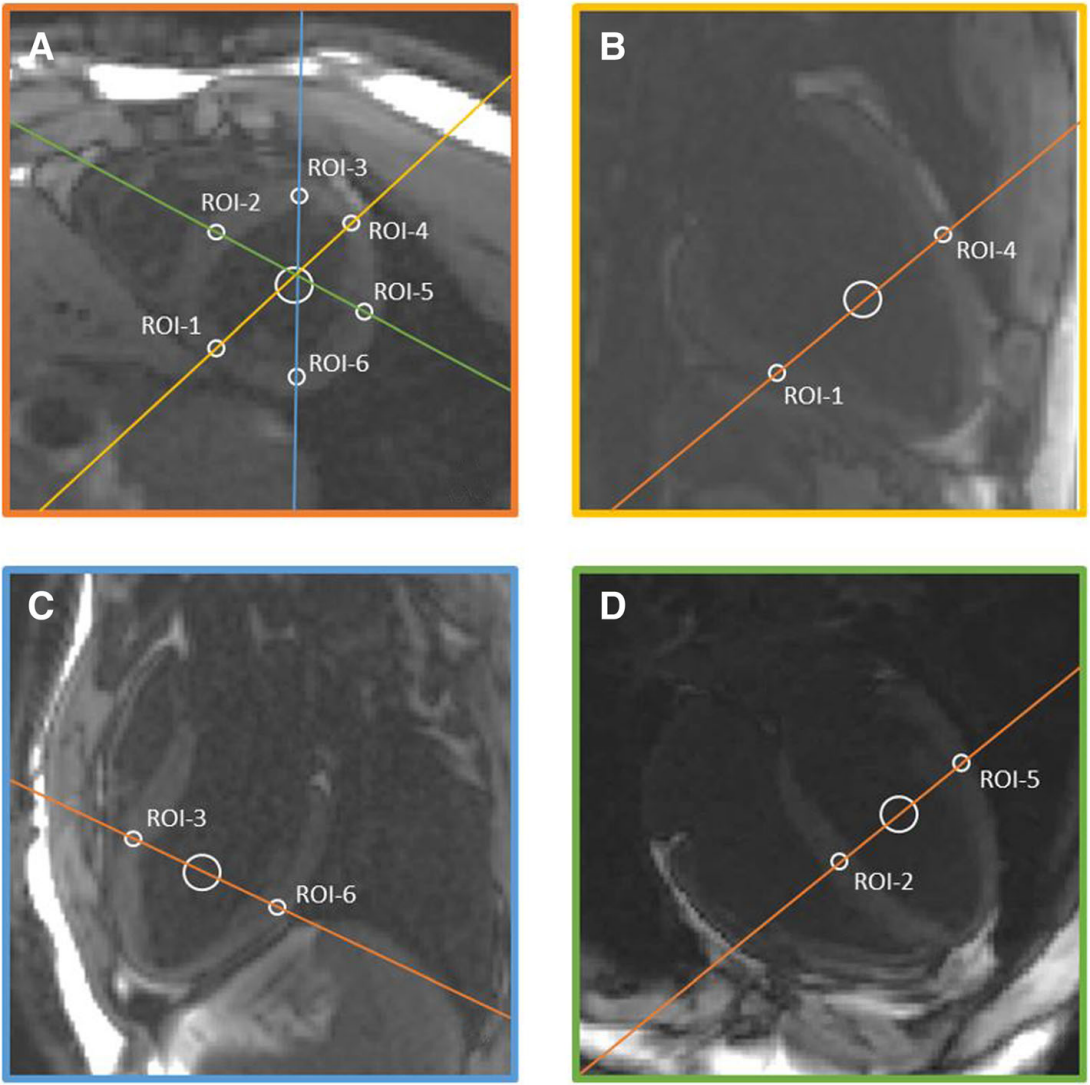
Myocardial and blood regions of interest on single-shot bSSFP images extracted from the MOLLI pulse sequence. **a** shows a midventricular short axis image, (**b**) shows a two-chamber long axis view image, (**c**) shows a three-chamber long axis view image and (**d**) shows a four-chamber long axis view image. The native T1 anatomical images (single-shot bSSFP images) have been extracted from the MOLLI pulse sequence for the time-point that presented the highest contrast between the blood pool and the myocardium. (bSSFP - Balanced Steady-State Free Precession, MOLLI - MOdified Look-Locker Inversion recovery)

The relaxation time parameters were estimated through a ROI-based curve fitting on the in-line, motion-corrected image series derived from both pre- and post-contrast MOLLI and SASHA acquisitions. All images were analyzed using the software Segment, version 2.0R5453 (http://segment.heiberg.se) [19]. T1 measurements were performed by drawing same size regions of interest (ROIs) on the myocardium (ROI area: 0.1 cm^2^) as well in the blood pool (ROI area: 0.8 cm^2^) at the intersections of the short axis and long axis images (Fig. 1), therefore the same tissue area was evaluated twice (once in short axis and once in long axis view). In healthy volunteers, the ROIs were placed at the center of the myocardial wall and special care was taken so as to avoid signal contamination from adjacent blood. In patients, ROIs of infarction and myocardium-at-risk (MaR) were considered as affected myocardium. Contrast enhanced SSFP (CE-SSFP) and late gadolinium enhancement (LGE) images were used to detect regional myocardial edema and fibrosis. Special care was taken so as to place the ROIs within a single tissue type area (remote, edema or fibrosis) and avoid signal contamination from adjacent tissue types. The placement of the ROIs was performed by an experienced reviewer (DN: 5 years of CMR experience). T1-values were measured both before and after a gadolinium (Gd) based contrast injection and myocardial ECV was calculated according to the following Eq. [1]:

1 $$ Myocardial\  ECV=\left(1- Hct\right)\frac{1/ Myocardial\ T{1}_{post\ contrast}-1/ Myocardial\ T{1}_{pre\  contrast}}{1/ Blood\ T{1}_{post\ contrast}-1/ Blood\ T{1}_{pre\  contrast}} $$

### Statistical analysis

Graphpad Prism version 7.03 (Graphpad Software Inc. La Jolla, USA) was used to perform statistical analysis. Values are presented as mean ± SD. Student’s two tailed t-test for paired data was utilized for comparison of different acquisitions. Differences with a *p*-value < 0.05 were considered to be statistically significant. Bland-Altman plots were also used to compare the short-axis native T1 and ECV values against the T1 values and ECV values extracted from the corresponding long-axis image.

## Results

### SAX vs LAX T1 and ECV values in healthy volunteers using MOLLI and SASHA

**Fig. 2:**
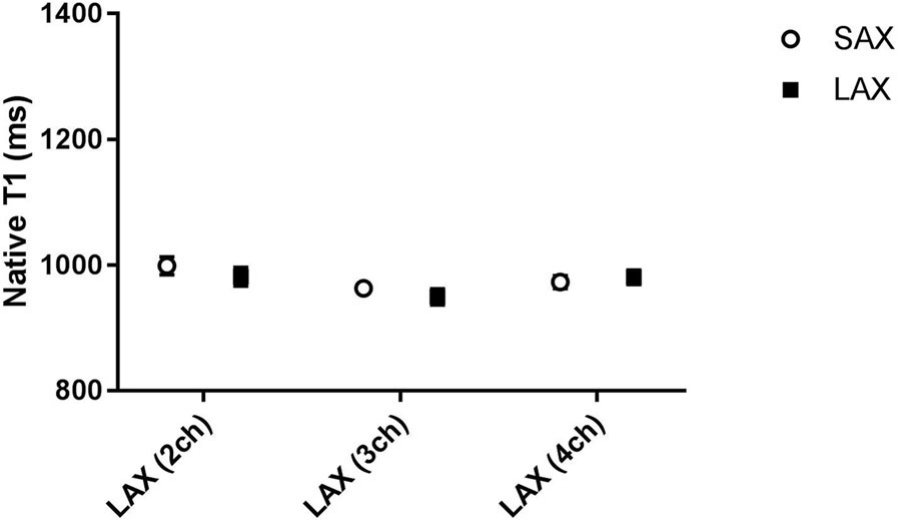
MOLLI T1 values in healthy volunteers – SAX vs. LAX: Mean native T1 values derived from ROIs placed in three LAX slices (2 chamber view, 3 chamber view and 4 chamber view – black rectangles) against the corresponding ROIs in the midventricular SAX plane (open circles) on a population of healthy volunteers. (ROI - Region of Interest, SAX - Short Axis, LAX - Long Axis)

**Fig. 3:**
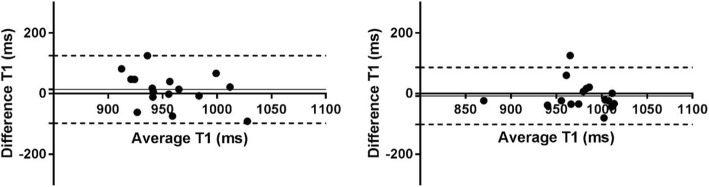
MOLLI native T1 values – SAX vs. LAX: Bland-Altman plots of the native T1 values obtained with MOLLI from ROIs placed in two LAX slices (3 chamber view on the left figure and 4 chamber view on the right figure) against the corresponding ROIs in the midventricular SAX plane on a population of healthy volunteers. Solid horizontal lines represent the means (bias), dashed horizontal lines represent the 95% confidence limits. (MOLLI - MOdified Look-Locker Inversion recovery, ROI - Region of Interest, SAX - Short Axis, LAX - Long Axis)

**Fig. 4:**
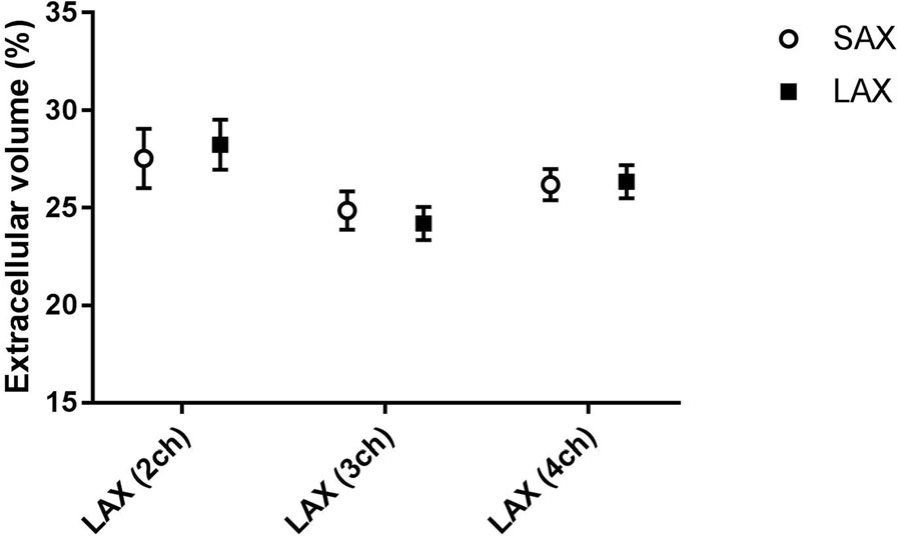
MOLLI ECV values in healthy volunteers – SAX vs. LAX: Mean myocardial ECV values derived from ROIs placed in three LAX slices (2 chamber view, 3 chamber view and 4 chamber view – black rectangles) against the corresponding ROIs in the midventricular SAX plane (open circles) on a population of healthy volunteers. (MOLLI - MOdified Look-Locker Inversion recovery, ROI - Region of Interest, SAX - Short Axis, LAX - Long Axis, ECV - Extracellular Volume)

**Fig. 5:**
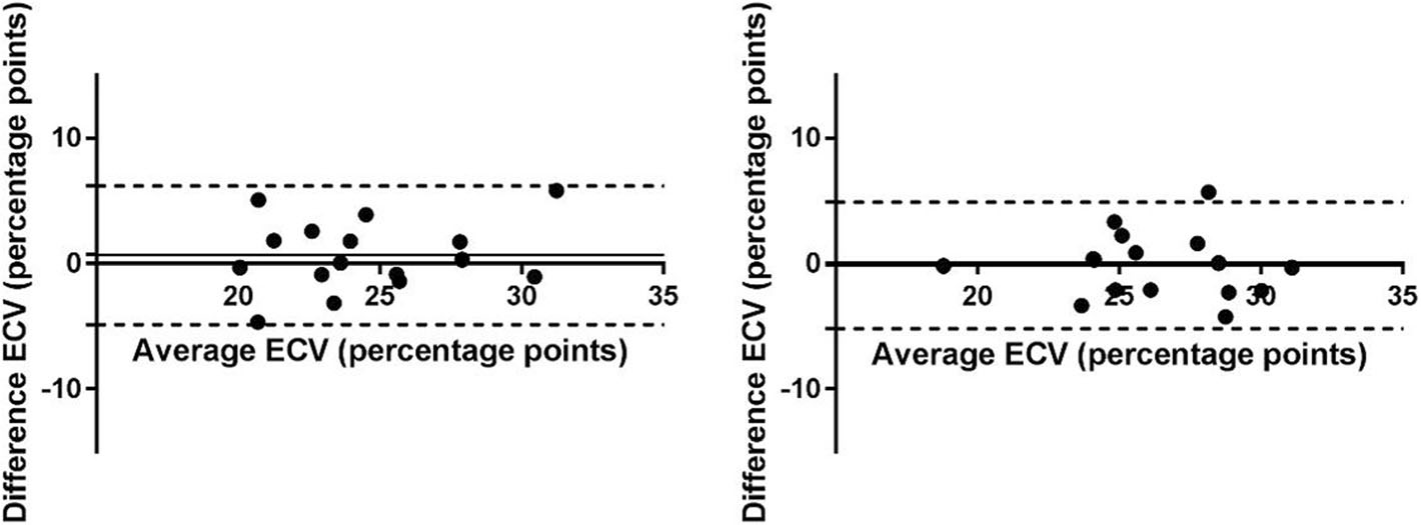
MOLLI ECV values – SAX vs. LAX: Bland-Altman plots of the myocardial ECV values obtained with MOLLI from ROIs placed in two LAX slices (3 chamber view on the left figure and 4 chamber view on the right figure) against the corresponding ROIs in the midventricular SAX plane on a population of healthy volunteers. Solid horizontal lines represent the means (bias), dashed horizontal lines represent the 95% confidence limits. (MOLLI - MOdified Look-Locker Inversion recovery, ROI - Region of Interest, SAX - Short Axis, LAX - Long Axis, ECV - Extracellular Volume)

In healthy volunteers, there were no statistically significant differences in native T1 and ECV values between short axis and long axis acquisitions (*p* > 0.05) using MOLLI acquisitions. Figure 2 presents the mean native T1 values derived from the ROIs placed in three LAX slices (2 chamber view, 3 chamber view and 4 chamber view) against the corresponding ROIs in the midventricular SAX plane. The mean native T1 values were 999 ± 58 ms vs. 982 ± 61 ms (SAX vs LAX 2ch, *n* = 16, *p* = 0.46), 963 ± 35 ms vs. 950 ± 51 ms (SAX vs LAX 3ch, n = 16, *p* = 0.35) and 973 ± 41 ms vs. 981 ± 46 ms (SAX vs LAX 4ch, *n* = 16, *p* = 0.53). The corresponding Bland-Altman plots for native T1 measurement in SAX view against the 3-chamber and 4 chamber LAX views are shown in Fig. 3. Figure 4 presents the mean ECV values derived from the ROIs placed in the same three LAX slices against the corresponding ROIs in the midventricular SAX plane. The mean ECV values were 27.5 ± 6.1% vs. 28.2 ± 5.1% (SAX vs LAX 2ch, *n* = 16, *p* = 0.59), 24.9 ± 4% vs. 24.2 ± 3.4% (SAX vs LAX 3ch, *n* = 16, *p* = 0.36) and 26.2 ± 3.2% vs. 26.3 ± 3.4% (SAX vs LAX 4ch, n = 16, *p* = 0.83). The corresponding Bland-Altman plots for ECV measurement in SAX view against the 3-chamber and 4 chamber LAX views are shown in Fig. 5.

**Fig. 6:**
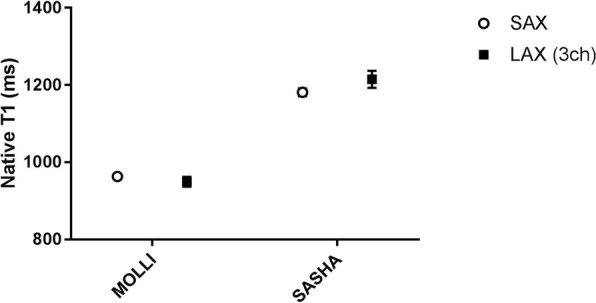
T1 values in healthy volunteers – MOLLI vs. SASHA: Mean native T1 values derived from ROIs placed in a three chamber LAX slice (black rectangles) against the corresponding ROIs in the midventricular SAX plane (open circles) with MOLLI (left) and SASHA (right) on a population of healthy volunteers. (MOLLI - MOdified Look-Locker Inversion recovery, ROI - Region of Interest, SAX - Short Axis, LAX - Long Axis, SASHA - Saturation recovery single-shot acquisition)

**Fig. 7:**
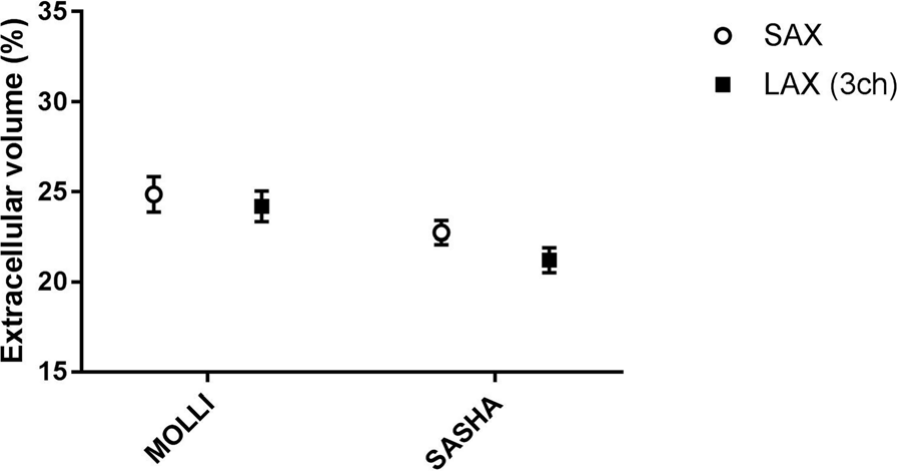
ECV values in healthy volunteers – MOLLI vs. SASHA: Mean myocardial ECV values derived from ROIs placed in a three chamber LAX slice (black rectangles) against the corresponding ROIs in the midventricular SAX plane (open circles) with MOLLI (left) and SASHA (right) on a population of healthy volunteers. (MOLLI - MOdified Look-Locker Inversion recovery, ROI - Region of Interest, SAX - Short Axis, LAX - Long Axis, SASHA - Saturation recovery single-shot acquisition, ECV - Extracellular Volume)

**Fig. 8:**
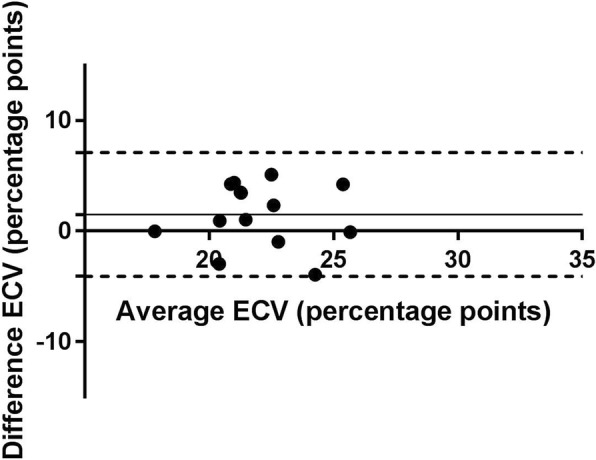
SASHA ECV values – SAX vs 3-chamber LAX: Bland-Altman plot of the myocardial ECV values obtained with SASHA from ROIs placed in three chamber LAX slice against the corresponding ROIs in the midventricular SAX plane on a population of healthy volunteers. Solid horizontal lines represent the means (bias), dashed horizontal lines represent the 95% confidence limits. (ROI - Region of Interest, SAX - Short Axis, LAX - Long Axis, SASHA - Saturation recovery single-shot acquisition, ECV - Extracellular Volume)

Native T1 and ECV measurements in SAX and 3-chamber LAX views using MOLLI and SASHA acquisitions are presented in Figs. 6 and 7 respectively. The mean native T1 values were 963 ± 35 ms vs. 950 ± 51 ms (SAX vs LAX 3ch, *n* = 16, *p* = 0.35) for MOLLI whereas the corresponding mean T1 values for SASHA were 1181 ± 38 ms vs. 1215 ± 83 ms (SAX vs LAX 3ch, *n* = 14, *p* = 0.13). The mean ECV values were 24.9 ± 4% vs. 24.2 ± 3.4% (SAX vs LAX 3ch, n = 16, *p* = 0.36) for MOLLI whereas the corresponding mean ECV values for SASHA were 22.8 ± 2.5% vs. 21.2 ± 2.6% (SAX vs LAX 3ch, n = 14, *p* = 0.07). The corresponding Bland-Altman plots for ECV measurement in SAX view against the 3-chamber LAX view using SASHA is shown in Fig. 8. In one volunteer data acquired using SASHA was excluded due to motion artifacts. No registration distortion [20] was observed in the motion corrected, T1-weighted image series derived from both MOLLI and SASHA acquisitions in healthy volunteers.

### SAX vs LAX T1 and ECV values in patients using MOLLI and SASHA

**Fig. 9:**
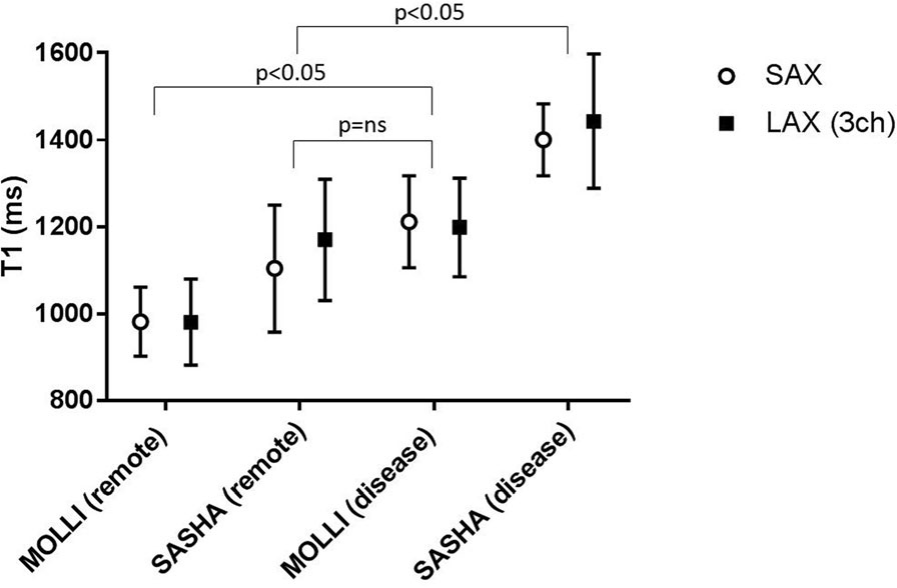
T1 values in patients – MOLLI vs. SASHA: Mean native T1 values derived from ROIs placed in remote and affected myocardium in a three chamber LAX slice (black rectangles) against the corresponding ROIs in the midventricular SAX plane (open circles) with MOLLI and SASHA on a population of patients. There was a statistically significant difference (*p* < 0.05) in T1 measurements between remote and diseased myocardium in both MOLLI and SASHA for both short axis and 3-chamber long axis acquisitions (MOLLI: SAX *p* = 0.0014, LAX *p* = 0.0026 – SASHA: SAX *p* = 0.0025, LAX *p* = 0.0019) However, there was no statistically significant difference (*p* > 0.05) between the MOLLI-based T1 measurements of diseased myocardium and the SASHA-based T1 measurements of remote myocardium for both short axis (*p* = 0.06) and 3-chamber long axis (*p* = 0.26) acquisitions. (MOLLI - MOdified Look-Locker Inversion recovery, ROI - Region of Interest, SAX - Short Axis, LAX - Long Axis, SASHA - Saturation recovery single-shot acquisition)

**Fig. 10:**
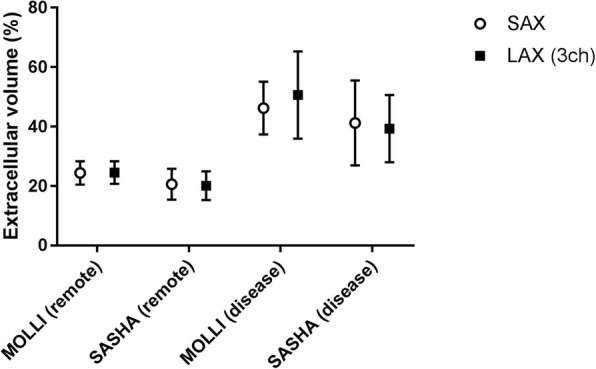
ECV values in patients – MOLLI vs. SASHA: Mean myocardial ECV values derived from ROIs placed in remote and affected myocardium in a three chamber LAX slice (black rectangles) against the corresponding ROIs in the midventricular SAX plane (open circles) with MOLLI and SASHA on a population of patients. (MOLLI - MOdified Look-Locker Inversion recovery, ROI - Region of Interest, SAX - Short Axis, LAX - Long Axis, SASHA - Saturation recovery single-shot acquisition, ECV - Extracellular Volume)

In patients, there were no statistically significant differences in native T1 and ECV values between short axis and 3-chamber long axis acquisitions (*p* > 0.05). The mean native T1 values extracted from ROIs placed in remote myocardium were 982 ± 79 ms vs. 981 ± 99 ms (SAX vs LAX 3ch, *n* = 7, *p* = 0.95) for MOLLI whereas the corresponding mean T1 values for SASHA were 1083 ± 147 ms vs. 1154 ± 145 ms (SAX vs LAX 3ch, *n* = 6, *p* = 0.14). The mean ECV values from the same ROIs placed in remote myocardium were 24.4 ± 3.9% vs. 24.6 ± 3.8% (SAX vs LAX 3ch, n = 7, *p* = 0.88) for MOLLI whereas the corresponding mean ECV values for SASHA were 20.2 ± 3.1% vs. 20.1 ± 5.3% (SAX vs LAX 3ch, n = 6, *p* = 0.94). In affected myocardium, the mean native T1 values were 1212 ± 106 ms vs. 1199 ± 113 ms (SAX vs LAX 3ch, *n* = 9, *p* = 0.48) for MOLLI whereas the corresponding mean T1 values for SASHA were 1407 ± 87 ms vs. 1416 ± 139 ms (SAX vs LAX 3ch, n = 9, *p* = 0.82). The mean ECV values from the same ROIs placed in affected myocardium were 46.2 ± 8.9% vs. 50.6 ± 14.7% (SAX vs LAX 3ch, n = 9, *p* = 0.28) for MOLLI whereas the corresponding mean ECV values for SASHA were 44.3 ± 11.7% vs. 41.3 ± 10.2% (SAX vs LAX 3ch, n = 9, *p* = 0.43). Figures 9 presents the mean T1 values extracted from remote and affected myocardium in SAX and 3-chamber LAX views using MOLLI and SASHA. The corresponding ECV values are shown in Fig. 10. In one patient data acquired using SASHA was excluded due to motion artifacts. No registration distortion [20] was observed in the motion corrected, T1-weighted image series derived from both MOLLI and SASHA acquisitions in patients.

Lastly, as Fig. 9 shows, there was a statistically significant difference (*p* < 0.05) in T1 measurements between remote and diseased myocardium in both MOLLI and SASHA for both short axis and 3-chamber long axis acquisitions (MOLLI: SAX *p* = 0.0014, LAX *p* = 0.0026 – SASHA: SAX *p* = 0.0025, LAX *p* = 0.0019). However, there was no statistically significant difference (*p* > 0.05) between the MOLLI-based T1 measurements of diseased myocardium and the SASHA-based T1 measurements of remote myocardium for both short axis (*p* = 0.06) and 3-chamber long axis (*p* = 0.26) acquisitions.

## Discussion

This study investigates T1 and ECV quantified from long-axis acquisitions compared to short-axis acquisitions using MOLLI and SASHA in healthy volunteers and patients. Long-axis acquisitions showed no statistically significant differences in native T1 and ECV values compared to short-axis acquisitions using both MOLLI and SASHA methods. The two-chamber long axis acquisition presented the lowest agreement with the short-axis acquisition compared to the other two long axis acquisitions. Both MOLLI and SASHA showed similarly tight limits of agreement when comparing the ECV measurements taken with short axis and three-chamber long axis acquisitions although SASHA showed larger bias than MOLLI. Lastly, the statistical hypothesis testing presented in Fig. 9 indicates that MOLLI and SASHA should not be used interchangeably in T1 mapping for characterizing different tissue types in myocardium.

In the last several years, CMR parametric mapping has been used as a non-invasive tool for quantifying myocardial tissue alternations in myocardial disease. Recently, the Society for Cardiovascular Magnetic Resonance (SCMR) endorsed by the European Association for Cardiovascular Imaging (EACVI) published a consensus statement with recommendations on T1 and ECV mapping [18]. The guidelines suggested that an optional single long axis map should be acquired in cases of global/diffuse diseases whereas acquisition of at least one long axis map is considered mandatory in cases of patchy diseases. Nowadays, MOLLI is mainly used as the preferred technique in cardiac T1 and ECV mapping [21] whereas SASHA has been proposed in the literature as a means of mitigating the T1-underestimation in MOLLI [7, 8]. However, there is limited work today in the literature that has evaluated the performance of T1 and ECV mapping in myocardial tissue characterization using long-axis against short-axis acquisitions. Moreover, although SASHA is actively being studied today, its performance in T1 and ECV measurements under different slice orientations has not been investigated before.

This study adds on the research that other groups have already performed in order to investigate the impact of slice position in CMR imaging. In particular, comparison of short and long axis methods had been the focus of other previous studies in CMR for volumetric measurements. Harizolan et al. [13] showed that there was no significant difference in both patient with myocardial infarction and control groups between end-diastolic volume determined from short axis slice and end-diastolic volume determined by 2-chamber, 3-chamber and 4-chamber long axis slices. In a more recent study, Huttin et al. [14] showed that measurements of left-ventricle volumes and ejection fraction utilizing a biplane long axis MRI study allowed for accurate, fast and reliable assessment of left-ventricle function and exhibited good correlation with the short axis measurements. Despite that the long axis acquisition presented a systematic underestimation of left-ventricles volumes compared to the short axis acquisition, the authors suggested that a biplane long axis MRI study should be considered to shorten the overall imaging acquisition in an acute clinical setting.

In the field of T1 and ECV mapping, results of the current work were similar to results previously reported in the literature. Nacif et al. [15] showed that there was no significant difference in global myocardial T1 values between four-chamber long-axis and mid-ventricular short-axis measurements using the MOLLI T1 mapping technique on a group of healthy volunteers. In another study, Bohnen et al. [16] compared global myocardial T1 and ECV values between short-axis and three different long-axis slices (two-chamber, three-chamber and four-chamber views) on patients with suspected myocarditis. Although the authors showed that there were no significant differences on ECV measurements between short and long axis slices, significantly lower median native myocardial T1 values on long axis slices were reported compared to short-axis slices. The latter was attributed to issues related to slice orientation (such as through-plane motion and partial volume effects) but also to the heterogeneity of myocardial injury in myocarditis. In the current study, although no significant differences on T1 and ECV measurements were shown between short and long axis slices using MOLLI, Figs. 2 and 4 presented a larger intra-slice variability of the MOLLI-based T1 and ECV estimates within the midventricular short axis image compared to the inter-slice variability (short axis vs long axis). In a similar manner, previous studies [11, 22, 23] have demonstrated significant regional variations of native T1 values in SAX slices of normal subjects. These differences were not considered representative of a true difference in tissue composition but were attributed to other factors, such as inadequate B0-shimming around the heart (off-resonance issues) [9], receiver coil sensitivity and distance of the receiver coil elements from the region of interest [24].

Compared to the previous two studies [15, 16], the current study did not measure the mean T1 and ECV values of the entire myocardium within the slice to evaluate the performance of long-axis acquisitions against the short-axis acquisitions. In this study, T1 measurements and ECV calculations were performed using an ROI-based analysis by drawing same size ROIs on the myocardium as well in the blood pool at the intersections of the short axis and long axis images. This approach was considered more representative for evaluating the differences between different slice orientations since it eliminates any T1 variability caused by biological focal abnormalities in the myocardium and enhances the investigation of any T1 variability caused by the technical design of the quantitative approach. In a similar approach, Caballeros et al. [17] showed that there was no significant difference in myocardial T1 values and ECV measurement between short-axis and four-chamber long axis analysis on groups of patients with various diseases.

### Limitations

In this study, some limitations apply. The number of subjects is small in both groups. Larger scale studies are required to detect potential subtle differences between different acquisition techniques in T1 and ECV measurements. Moreover, the design of the current study does not allow for a direct comparison between MOLLI and SASHA on the inter-slice variability of the T1 and ECV estimates using the two-chamber and four-chamber LAX images since these images were not acquired neither in healthy volunteers nor in patients. Lastly, the post contrast myocardial T1 values were not presented in this study. Post contrast T1 mapping is considered more variable and depend on several factors such as time elapsed between contrast agent administration and renal clearance [25].

## Conclusions

In conclusion, long axis measurements of myocardial T1 and ECV using MOLLI and SASHA exhibit good agreement with the corresponding short axis measurements allowing for fast and reliable myocardial tissue characterization. This may be of high importance in clinical cases where shortening of the overall imaging acquisition is required. Moreover, the ROI-based design of the current study may be utilized in other studies that are focused on myocardial tissue characterization in order to evaluate the differences between different slice orientations, especially in cases with focal native T1 abnormalities.

## Abbreviations

bSSFP: Balanced Steady-State Free Precession
CMR: Cardiovascular Magnetic Resonance
ECV: Extracellular volume
LAX: Long axis
MOLLI: MOdified Look-Locker Inversion recovery
ROI: Region of Interest
SASHA: Saturation recovery single-shot acquisition
SAX: Short axis
